# Incidence of chronic myeloid leukemia in Calgary, Alberta, Canada

**DOI:** 10.1186/s13104-018-3890-8

**Published:** 2018-11-01

**Authors:** Leonard Tu Nguyen, Maggie Guo, Christopher Naugler, Fariborz Rashid-Kolvear

**Affiliations:** 1 Department of Pathology and Laboratory Medicine, Cumming School of Medicine, University of Calgary, Calgary, AB Canada; 2 Departments of Family Medicine and Community Health, Cumming School of Medicine, University of Calgary, Calgary, AB Canada; 3 Department of Medical Genetics, Cumming School of Medicine, University of Calgary, Calgary, AB Canada; 4 Diagnostic and Scientific Centre, 2E-415, 9 3535 Research Road NW, Calgary, AB T2L2K8 Canada

**Keywords:** Chronic myeloid leukemia, Chronic myelogenous leukemia, Myeloproliferative neoplasm, Cancer epidemiology, Hematological neoplasm, incidence, Canada

## Abstract

**Objective:**

The epidemiology of chronic myeloid leukemia is shifting due to the aging global population and the recent discovery and availability of targeted treatment options. This study provides recent data regarding the incidence of CML in Calgary, a major Canadian city. Data from patients diagnosed with CML by bone marrow sample analysis from 2011 to 2015 were collected from the database of the sole centralized cytogenetics facility in service of Calgary and its surrounding area.

**Results:**

With an average of 10.2 newly diagnosed cases per year in Calgary from 2011 to 2015, the incidence rate was calculated to be 0.75 cases per 100,000 person-years (95% CI 0.57–0.99). With age standardization, the incidence was 0.87 cases per 100,000 person-years (95% CI 0.82–0.91) for the Canadian population, which was low compared to other developed Western nations. The highest incidence rates were observed in the older patient categories, however there was a broad age distribution for incident cases and the median age at diagnosis was 48. There was a general male bias for CML most pronounced at the younger ages. Our description of CML incidence will help to inform healthcare planners amidst the dramatically altered treatment of this hematological neoplasm.

## Introduction

Chronic myeloid leukemia (CML) is a progressive clonal myeloproliferative disorder that accounts for roughly 15% of adult leukemias [1]. Most diagnoses occur in asymptomatic patients in the chronic stage, with a small proportion of cases having progressed to the accelerated phase or blast crisis depending on the blast count [2]. CML is more predominant in males than females and it is generally described to occur later in life, however the median age of diagnosis differs between cancer registries from the late 30’s in low and middle income countries up to 65 [3, 4]. There is no strong evidence for bias towards particular ethnicities despite lower incidence rates reported in several Asian countries and populations [5, 6]. Other associated risk factors include ionizing radiation, as most famously illustrated in Japanese atomic bomb survivors [7], and recently recognized lifestyle factors including smoking and body mass [8, 9].

The pathogenesis of CML stems from a distinct chromosomal translocation, named the Philadelphia (Ph) chromosome, which fuses the bcr and abl gene sequences from chromosomes 22 and 9, respectively [10]. The expressed Bcr–Abl chimeric product is an oncoprotein with constitutive tyrosine kinase activity, leading to the proliferation of myeloid cells in the bone marrow. There are rare cases of Ph-negative or atypical CML presenting with the same histopathological clinical features as BCR–ABL1 positive CML such as an elevated granulocyte count in a CBC and high proportion of myelocytes in a peripheral blood smear [11]. Otherwise, the Ph chromosome is detected by routine karyotyping in a cytogenetics facility, with FISH or RT-PCR as supporting techniques to show evidence of the translocation. The management of CML progressed extensively after imatinib was first described in 1996 as a tyrosine kinase inhibitor (TKI) specific to BCR–ABL1 [12, 13]. Since then, second- and third-generation TKIs have been developed as alternatives for patients who have become resistant to imatinib or had poor tolerability [14]. With these highly targeted treatment options, CML survival rates have improved drastically often to give a life expectancy approaching that of the general population [15]. Concurrently, its prevalence has increased in the last decade and the requirement of continuous TKI therapy in most cases will have an impact on future healthcare spending [6].

Here, we describe the incidence of CML in Calgary, a major Canadian city, for the recent years of 2011–2015. This will add to the global literature of epidemiological data for CML and aid clinician awareness as early diagnoses in the chronic phase with low BCR–ABL1 levels and blast counts will increase the likelihood of patients’ successful response to treatment [16].

## Main text

### Methods

#### Ethics approval

This study was approved by the Health Research Ethics Board of the Alberta Cancer Committee (ID HREBA.CC-16-0830).

#### Data source

Patient data for this retrospective observational study were obtained from the Calgary Laboratory Services (CLS) Cancer Cytogenetics Laboratory, which receives bone marrow samples from the city of Calgary and the surrounding Southern Alberta area. In accordance with the 2008 WHO guidelines for diagnosis [17], suspected CML cases from abnormal blood counts are followed by bone marrow biopsy for chromosomal banding analysis and/or fluorescence in situ hybridization to identify the t(9;22)(q34.1;q11.2) translocation. Newly diagnosed cases within the study period of January 1, 2011 to December 31, 2015 underwent postal code verification to exclude patients living outside the City of Calgary.

#### Case identification

Incident cases of CML were categorized by age and gender, and incidence rates were calculated with 95% confidence intervals. Calgary population data used in the calculations were taken from Statistics Canada in the form of updated population estimates determined by post-censal coverage studies [18]. Direct age standardization of the crude CMA incidence rate to the Canadian 2011–2015 population was calculated using published methods [19] and age-categorized population estimates from Statistics Canada [20]. 95% confidence intervals were calculated for each incidence rate by Wilson score interval for binomial proportions with sample sizes of 6,775,552 and 175,645,187 representing cumulative 2011–2015 population totals for Calgary and Canada, respectively.

### Results

**Fig. 1:**
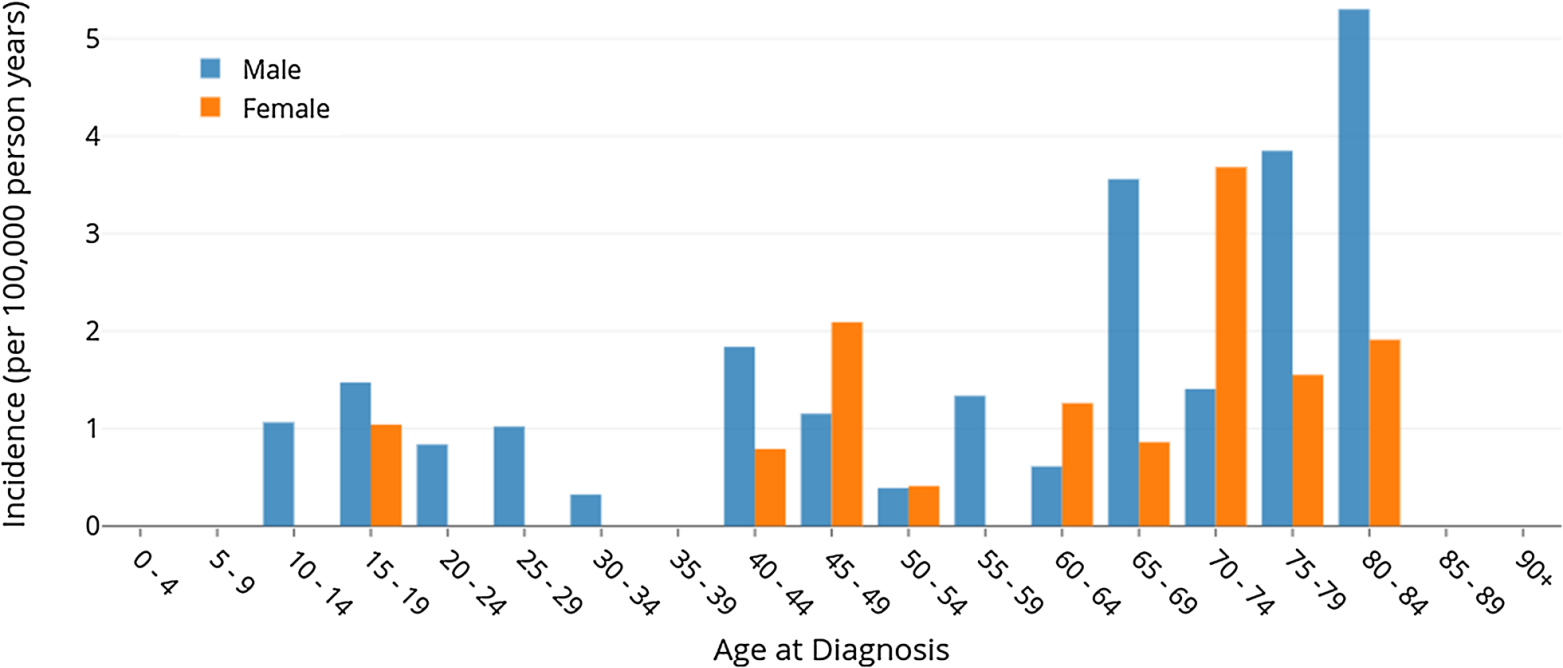
Age- and gender-stratified incidence of chronic myeloid leukemia in Calgary from 2011 to 2015

With an average 10.2 new cases over 2011–2015, the crude incidence of CML in Calgary was 0.75 cases per 100,000 person-years (95% CI 0.57–0.99) (Table 1). With age-standardization to the Canadian population, the incidence was 0.87 cases per 100,000 person-years (95% CI 0.82–0.91). 16 cases of Ph+ CML were observed for every Ph− CML. The strong male preference (1.83:1M:F) was reflected in the age distribution of incident cases (Fig. 1), which showed a broad distribution beginning in the early teenage years and peaking in the late 70–74 and 80–84 age categories for females and males, respectively.

**Table 1:** Incidence features of chronic myeloid leukemia in Calgary (2011–2015)

Total new cases (per year)	10.2
Ph+	9.6
Atypical CML, Ph−	0.6
Crude incidence rate (per 100,000 person-years)	0.75
95% confidence intervals	(0.57–0.99)
Canadian incidence rate (age-standardized, per 100,000 person-years)	0.87
95% confidence intervals	(0.82–0.91)
Male/female	1.83
Median age of diagnosis	48
Age range	11–84

### Discussion

Given the breakthrough developments in available therapy, ongoing refinements in diagnostic criteria and aging populations, the epidemiology of CML is radically changing [6]. Our results describing the recent incidence of CML in Calgary, a major Canadian city with a catchment population of 1.4 million, contributes to the monitoring of these shifting statistics and serves as an indication for healthcare planners in their future management of patients and resources. Obtaining accurate counts of cancer cases is a challenge that is continuously faced by registries around the world [21].

The age-standardized incidence rate of 0.87 CML cases per 100,000 person-years in Canada is considerably higher than the crude incidence calculated for Calgary, which is a reflection of the relatively younger population in the latter. These rates are low compared to other Western nations and more alarmingly, they are roughly half that of the United States (Table 2). The median age at diagnosis of 48 is also considerably lower compared to the United States and Western Europe. Instead, it is closer to the developed Asian populations of Hong Kong and Singapore. This is also likely due to the young population of Calgary, and higher incidence is still largely observed in the elderly from 65 to 84 years old (Fig. 1). Low incidence rates and ages at diagnosis are usually associated with low and middle income countries where there may be possible environmental factors and underreporting [4], however these causes are unlikely to be applicable here. The 6% incidence of atypical CML (Ph−) is higher than the generally accepted rate of 1–2% [11].

**Table 2:** Incidence rates per 100,000 person-years, male to female ratio, and median age of diagnosis in select global populations

Region	Period of study	Incidence (per 100,000 person-years)	M/F	Median age	Source
Calgary, AB	2011–2015	0.75	1.83	48	This paper
Canada^a^		0.87	–	–	This paper
United States	1975–2009	1.75	1.73	65	SEER [23]
Europe	2000–2002	1.1	1.25	–	[24]
Spain	2010–2012	1.08	1.61	54	[25]
United Kingdom	2004–2009	0.9	1.48	59	[26]
Lithuania	2000–2013	1.28	1.25	62	[27]
Hong Kong	1983–2003	0.9	1.5	49	[5]
Singapore	1998–2002	0.7	1.3	43	[5]
Russia	2009–2012	0.58	–	–	[28]
India	1976–2005	0.71 (M)/0.53 (F)	1.6	35	[29]

*SEER* surveillance, epidemiology, and end results

^a^Standardized rate from a population subset

Within the 5-year study period in Calgary, there is a strong pattern of new CML diagnoses in males over females that is higher than observed in other countries. This is most pronounced at young patient ages below 45 years old where male cases outnumber female cases by 4:1. For older patients, the male-to-female ratio is reduced to 1.21:1 and in a few of these mid-to-late age categories, there are higher female incidence rates of CML than males. Hematological neoplasms are usually more common in males than females due to genetic and hormonal differences [22].

In conclusion, we report a relatively low incidence of 0.75 cases of chronic myeloid leukemia per 100,000 person-years in Calgary, Canada from 2011 to 2015 (95% CI 0.57–0.99). The median age at diagnosis is also younger compared to other developed Western nations, and a strong male bias is observed.

## Limitations

The data presented here were limited by the geography and study period being considered, which skews the cohort towards a young, nonsmoking urban population. Furthermore, the summary statistics do not specify incident CML cases by phase. However, the low number of reported cases is less likely to provide accurate statistics for such classification. Future studies may also include cohort stratification by sociodemographic characteristics such as ethnicity or lifestyle factors, and consider other updated epidemiological measures such as survival rates and prevalence for CML in the TKI era.

## Abbreviations

CLS: Calgary Laboratory Services
CML: chronic myeloid leukemia
Ph: Philadelphia chromosome
TKI: tyrosine kinase inhibitor
